# Long-term significant seasonal differences in the numbers of new-borns with an orofacial cleft in the Czech Republic – a retrospective study

**DOI:** 10.1186/s12884-018-1981-0

**Published:** 2018-08-28

**Authors:** Miroslav Peterka, Zbynek Likovsky, Ales Panczak, Renata Peterkova

**Affiliations:** 1 Department of Teratology, Institute of Experimental Medicine, Czech Academy of Sciences, Videnska 1083, 142 20 Prague 4, Czech Republic; 2 Cleft Centre, Plastic Surgery Clinic at Kralovske Vinohrady Hospital in Prague, Prague, Czech Republic

**Keywords:** Orofacial cleft, Cleft lip, Cleft palate, Birth rate, New-born, Inborn defect, Seasonality, Variation, Warm season

## Abstract

**Background:**

Choosing the optimal season for conception is a part of family planning since it can positively influence the pregnancy outcome. Changes in the monthly number of infants born with a birth defect can signal prenatal damage - death or malformation – related to a harmful seasonal factor. The aim of our paper was to search for possible seasonal differences in the numbers of new-borns with an orofacial cleft and thus for a period of conception that can increase the risk of orofacial cleft development.

**Methods:**

Mean monthly numbers of live births in the Bohemia region of the Czech Republic during the years 1964–2000 were compared within a group of 5619 new-borns with various types of orofacial clefts and the control group derived from natality data on 3,080,891 new-borns.

**Results:**

The control group exhibited regular seasonal variation in the monthly numbers of new-borns: significantly more babies born during March–May and fewer babies born during October–December. Similar natural seasonal variation was also found in the group of babies with an orofacial cleft. However, after subdividing the cleft group according to gender and cleft type, in comparison to controls, significant differences appeared in the number of new-born girls with cleft lip during January–March and in the number of boys born with cleft palate in April – May.

**Conclusions:**

We found significant differences from controls in the number of new-born girls with CL and boys with CP, whose dates of birth correspond to conception from April to August and to the estimated prenatal critical period for cleft formation from May to October. The latter period includes the warm season, when various injurious physical, chemical and biological factors may act on a pregnant woman. This finding should be considered in pregnancy planning. Future studies are necessary to investigate the putative injurious factors during the warm season that can influence pregnancy outcome.

**Electronic supplementary material:**

The online version of this article (10.1186/s12884-018-1981-0) contains supplementary material, which is available to authorized users.

## Background

At the population level, birth seasonality is a complex phenomenon that is influenced by rhythmical environmental factors, such as photoperiods [1, 2] and seasonally high temperatures, which reduce spermatogenesis, ovulation and early embryo survival [3] and/or the availability of nutrition [2]. These factors result in seasonal differences in natality that can be specific for each country [3] and that can be influenced by a specific nation’s culture [4] and socio-demographic factors [5]. The Czech Republic is a central European country with a moderate climate and 4 distinct seasons (spring, summer, autumn and winter). There is a stable seasonality in the total number of births in the Bohemia region of the Czech Republic with a baby boom in the spring and a decrease in autumn [6].

Seasonal variation also occurs in the number of congenital anomalies, including orofacial clefts [7–9]. Seasonal variability in the occurrence of orofacial clefts has been reported, e.g., in Scotland [10], England [11], the United States [12], Zambia [13], Puerto Rico [14], Finland [15] and China [16]. In contrast, several studies have not confirmed the existence of seasonal variability of orofacial clefts, for example, in Georgia, USA [17], Colorado, USA [18], Northern Ireland [19] and Germany [20].

**Fig. 1:**
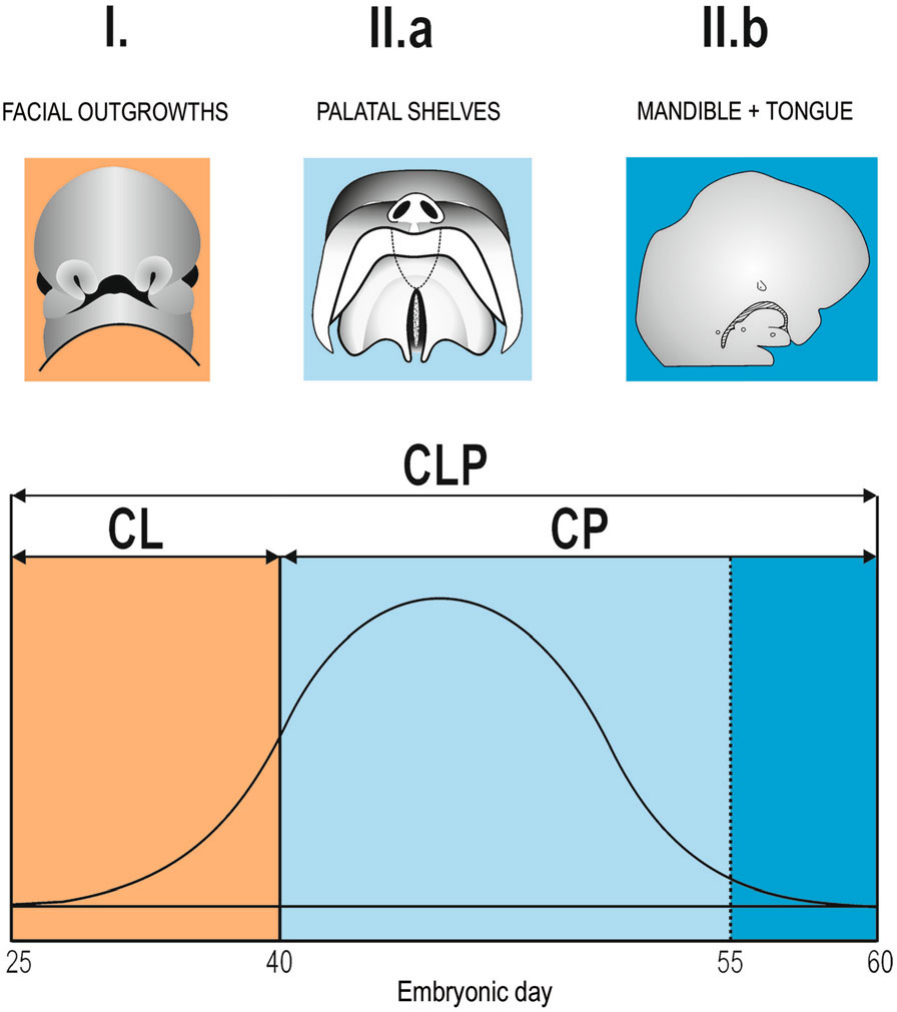
Critical period for orofacial cleft formation in humans. During critical period I (approximately embryonic days 30–40), cleft lip and jaw (CL) can be induced by a harmful environmental factor. During critical period II, cleft palate (CP) develops as a result of either hypoplasia of the palatal shelves (period II.a – approximately embryonic day 40–55) and/or by retarded growth of the mandible (period II.b – approximately embryonic day 55–60). As a result of its retarded forward growth, the short mandible does not withdraw the tongue from the space between the palatal shelves and so prevents their horizontalization. The total cleft lip and palate (CLP) develops during the critical period of CL and CP. (Adapted according to [63])

Orofacial clefts have a complex aetiology including multiple epigenetic (environmental) and genetic factors [21, 22]. The most common are non-syndromic clefts, which are likely due to genetic-environmental interactions [23]. Some of the risky environmental factors also exhibit seasonal variations. For example, seasonal variation in the incidence of orofacial clefts has been correlated with temperature changes according to climatic regions [12]. Chung et al. [24] have found a correlation between the monthly numbers of new-borns with orofacial clefts and the average monthly levels of sunshine, nitrogen oxide and nitrogen monoxide. There are also some additional factors that exhibit seasonal variability and can increase the risk of clefts in new-borns, such as the availability of specific nutrients (e.g., a deficiency of folic acid and/or other vitamins) [25–27], contamination by agriculture chemicals such as pesticides, and cosmic or other radiation [12]. Harmful environmental factors can induce an orofacial cleft in human embryos, provided they act during the critical period of development – between embryonic days 30–60, when the orofacial processes fuse to form a continuous upper lip and palate (Fig. 1).

We investigated the monthly birth rate of children with various types of orofacial clefts and that of all new-borns in the Bohemia region of the Czech Republic during 1964–2000. The aim of our study was to search for possible seasonal differences in the numbers of new-borns with an orofacial cleft during the years 1964–2000 and thus for a period of conception that can increase the risk of orofacial cleft development.

## Methods

In the Bohemia region of the Czech Republic, the treatment of patients with an orofacial cleft is centred at the national Cleft Centre at the Clinic of Plastic Surgery in Prague. We extracted anonymous birth rate data from the complete collection of history records, which was available on all 5619 children (3216 boys and 2403 girls) who were born with an orofacial cleft in the Bohemia region of the Czech Republic from 1964 to 2000 and treated at the Cleft Centre. The years 1964–2000 match the period when the treatment of the patients was centralized at the Cleft Centre and the patients´ register was complete.

It is known that there are gender differences in the prevalence of various cleft types; CP occurs more often in girls, while CL and CLP occur more often in boys (e.g., [28]), which has also been confirmed in the Czech population [29]. Therefore, for a more detailed analysis, we subdivided the group of cleft patients according to their gender and cleft type. Three cleft groups were considered: 1) group CP - children with an isolated cleft palate; 2) group CL - children with an isolated cleft lip; and 3) group CLP - children with a combined cleft lip and palate. The patients in each group were then grouped according to the month and year of their birth.

For comparison of the birth rate in the patients, national data were used on the total number of live new-borns in each month and year in the Bohemia region of the Czech Republic during the period 1964–2000, including 3,080,891 live-born children (1,582,625 boys and 1,498,266 girls). These anonymous data were obtained from the official database of the Czech Statistical Office [30]. For comparison of the birth rate between the cleft patients and controls, the control group (including only the new-borns without an orofacial cleft) was formed by subtracting the cleft patients from the total number of live new-borns.

### Statistical evaluation

The differences in total numbers of children born in a particular month of a year during the period of 37 years (**Σ** in the Tables 1 and 2) were tested between the control group and cleft (CL, CLP, CP) groups. Fisher’s exact test [31] was used, which allowed for the comparison of different size samples (large control group versus smaller cleft groups, in our case).

**Table 1:** Boys born during 1964–2000

Month		I	II	III	IV	V	VI	VII	VIII	IX	X	XI	XII
NC	Σ	221,358	208,245	244,215	241,422	220,682	229,161	230,717	217,741	213,307	203,066	193,035	199,661
	N	37	37	37	37	37	37	37	37	37	37	37	37
	x̄	5982.65	5628,24	6600.41	6524.92	5964.38	6193.54	6235.59	5884.89	5765.05	5488.27	5217.16	5396.24
	SE	198.91	201.76	245.50	241.65	374.36	209.70	203.04	185.84	192.77	188.69	178.70	195.72
CL	Σ	74	77	72	67	71	72	64	88	69	68	54	56
	N	37	37	37	37	37	37	37	37	37	37	37	37
	x̄	2.00	2.08	1.95	1.81	1.92	1.95	1.73	2.38	1.86	1.84	1.46	1.51
	SE	0.22	0.25	0.25	0.21	0.24	0.22	0.23	0.30	0.23	0.23	0.20	0.20
CLP	Σ	108	129	132	133	155	142	133	138	102	98	109	118
	N	37	37	37	37	37	37	37	37	37	37	37	37
	x̄	2.92	3.49	3.57	3.59	4.19	3.84	3.59	3.73	2.76	2.65	2.95	3.19
	SE	0.28	0.38	0.30	0.32	0.38	0.36	0.30	0.36	0.26	0.34	0.31	0.33
CP	Σ	75	70	78	60	98	70	85	72	71	77	61	64
	N	37	37	37	37	37	37	37	37	37	37	37	37
	x̄	2.03	1.89	2.11	1.62	2.65	1.89	2.30	1.95	1.92	2.08	1.65	1.73
	SE	0.27	0.26	0.22	0.22	0.32	0.25	0.20	0.22	0.25	0.26	0.23	0.21

Groups of boys: *NC* non-cleft control, *CL* cleft lip, *CLP* cleft lip and palate, *CP* cleft palate

**Σ -** total monthly number corresponding to all boys that born in a given month for 37 years; N – number of years; x̄ – mean monthly number of newborn boys;

SE – standard error of the mean

**Table 2:** Girls born during 1964–2000

Month		I	II	III	IV	V	VI	VII	VIII	IX	X	XI	XII
NC	Σ	207,032	198,352	230,452	229,166	230,680	216,559	218,629	207,749	201,606	192,596	182,260	189,343
	N	37	37	37	37	37	37	37	37	37	37	37	37
	x̄	5595.46	5360.86	6228.43	6193.68	6234.59	5852.95	5908.89	5614.84	5448.81	5205.30	4925.95	5117.38
	SE	196.73	192.24	230.57	229.77	223.85	196.62	192.30	191.31	183.63	177.38	172.90	181.12
CL	Σ	29	36	61	58	41	46	44	39	45	35	36	40
	N	37	37	37	37	37	37	37	37	37	37	37	37
	x̄	0.78	0.97	1.65	1.57	1.11	1.24	1.19	1.05	1.22	0.95	0.97	1.08
	SE	0.13	0.15	0.26	0.22	0.18	0.18	0.17	0.18	0.18	0.14	0.17	0.20
CLP	Σ	60	68	68	64	73	65	60	52	63	48	50	56
	N	37	37	37	37	37	37	37	37	37	37	37	37
	x̄	1.62	1.84	1.84	1.73	1.97	1.76	1.62	1.41	1.70	1.30	1.35	1.51
	SE	0.24	0.27	0.20	0.20	0.23	0.21	0.20	0.21	0.25	0.19	0.20	0.22
CP	Σ	98	96	107	104	101	98	111	91	91	93	84	92
	N	37	37	37	37	37	37	37	37	37	37	37	37
	x̄	2.65	2.59	2.89	2.81	2.73	2.65	3.00	2.46	2.46	2.51	2.27	2.49
	SE	0.25	0.30	0.31	0.32	0.31	0.28	0.24	0.28	0.28	0.25	0.24	0.30

Groups of girls: *NC* non-cleft control, *CL* cleft lip, *CLP* cleft lip and palate, *CP* cleft palate

**Σ -** total monthly number corresponding to all girls that born in a given month for 37 years; N – number of years; x̄ – mean monthly number of newborn girls; SE – standard error of the mean

Thereafter, the data on all the cleft and control groups were treated in the same way: for each particular month in the year, the mean monthly number of new-borns ($\overline{x}$**)** was calculated from the total number of infants born in this month during the whole period 1964–2000 (Tables 1 and 2). Special tests recommended appraising seasonal variation in descriptive epidemiology (Freedman’s test for monthly data to detect departures from a uniform occurrence throughout the year; Edward’s test to determine the amplitude of the curve, the time of the peak; the Ratchet circular scan test to detect significant peak periods, and Hewitt’s rank-sum test to detect significant peak periods) were used [32] to test the seasonal variation of the mean number of new-borns in each particular month of the year in the control groups and cleft groups ($\overline{x}$ in the Tables 1 and 2).

**Fig. 2:**
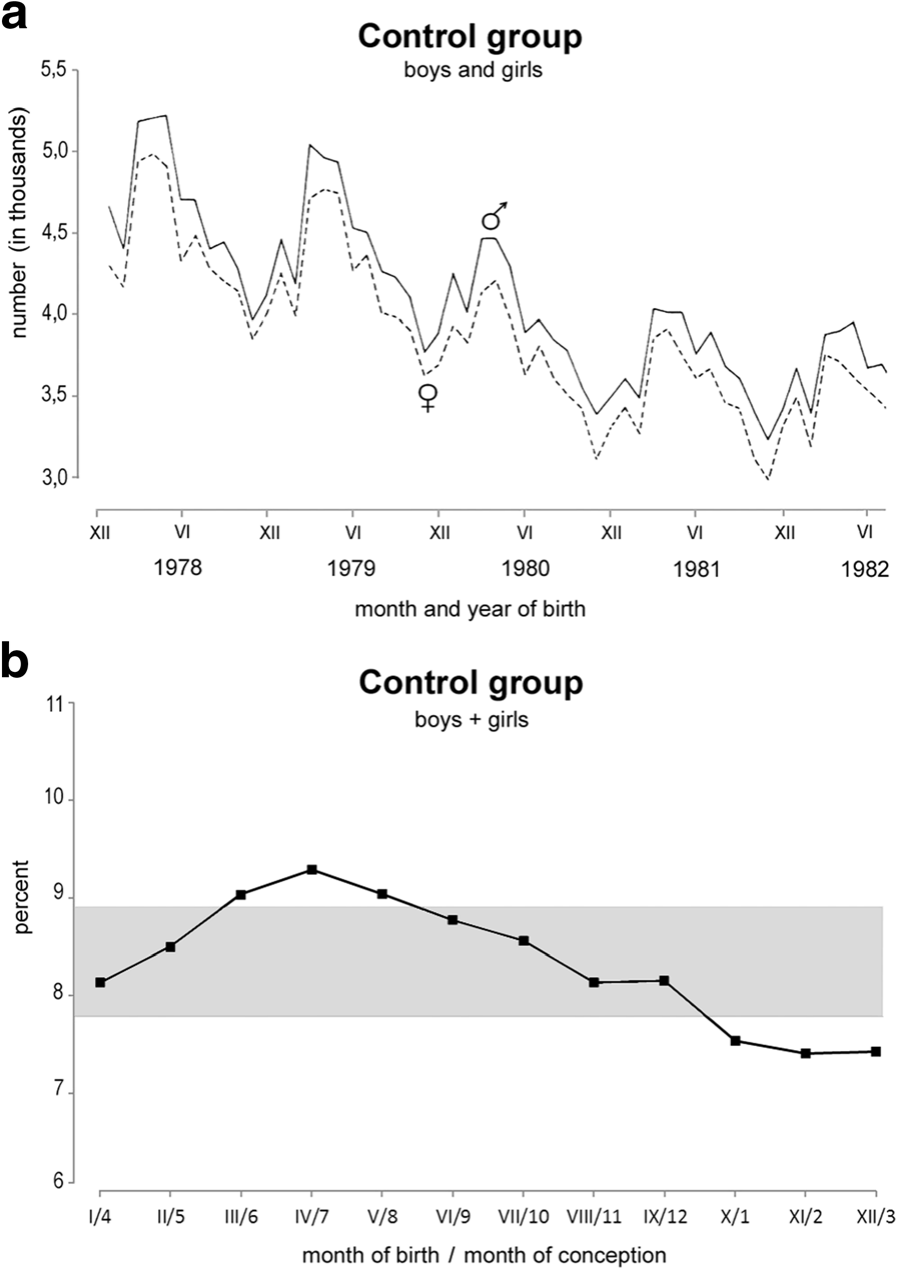
Monthly birth rate in the Czech population. **a** Number of new-born boys and girls per month during 1978–1982. Solid line – boys, dashed line – girls. **b** The percent of the mean monthly number of new-born girls + boys of a control group through 1964–2000. The horizontal lines represent the upper and lower limit of a 95% confidence interval indicated here by a grey strip. The confidence interval comprises the values that do not significantly differ from the mean value. The data located above or below the grey 95% interval are significantly (*p* < 0.05) higher or lower than the mean value, respectively. A significant baby boom was found in March, April, and May. Such delivery timing corresponds to conception during the summer time - in June, July, and August. A significant minimum of new-borns was observed in October, November, and December, corresponding to conception during the winter time - in January, February and March. Axis x: Roman digit - month of delivery, Arabic numeral - month of conception

The mean daily number of new-borns in each month (see below) were only used for testing the significance of the seasonal variation in the control group by the Confidence interval test [33] (Fig. 2b).

### Graphical presentation

**Fig. 3:**
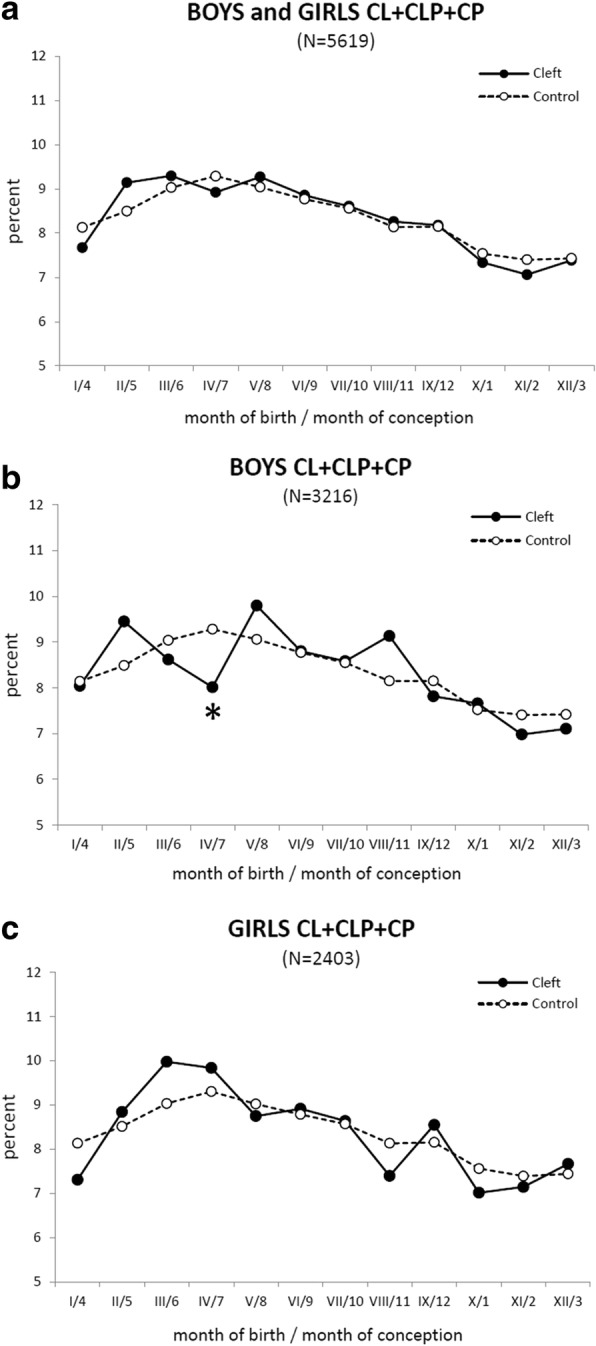
The mean daily number of new-borns in each month. The mean daily number (MDN) in percent of new-borns with an orofacial cleft (solid line) is compared with the control group (dashed line). **a** All girls + boys with cleft lip and jaw (CL), cleft lip and palate (CLP), and cleft palate (CP). **b** The boys with orofacial clefts: CL, CLP, and CP. **c** The girls with orofacial clefts: CL, CLP, and CP. Axis x: Roman digit - month of delivery, Arabic numeral - month of conception. Asterisk (*) shows the position of a decline (B) corresponding to a statistically significant (*p* < 0.05) decrease detected by Fisher’s exact test [31] in the total monthly number of new-born boys with an orofacial cleft

**Fig. 4:**
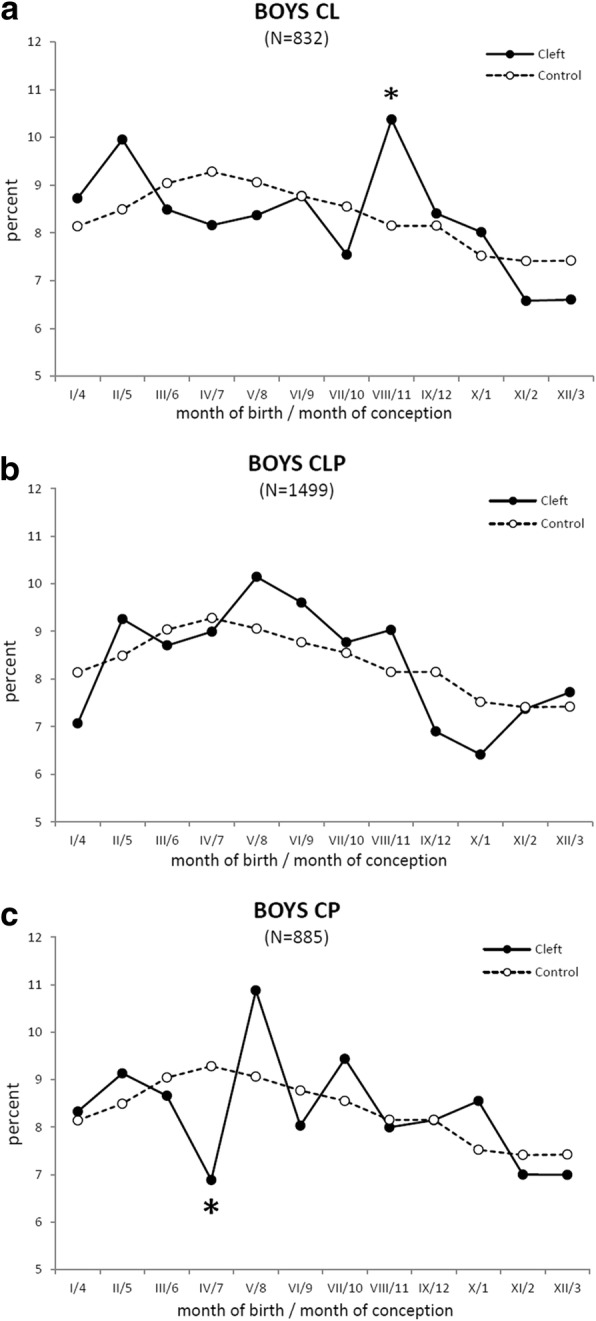
The mean daily number of new-born boys in each month. The mean daily number (MDN) in the percent of new-born boys with an orofacial cleft (solid line) is compared with the control group of boys (dashed line). **a** The boys with cleft lip and jaw (CL). **b**. The boys with cleft lip and palate (CLP). **c** The boys with cleft palate (CP). Axis x: Roman digit - month of delivery, Arabic numeral - month of conception. Asterisk (*) shows the position of a peak (A) or decline (C), corresponding to a statistically significant increase (*p* < 0.03) in the total monthly number of new-born boys with CL for 37 years (A) or to a significant decrease (*p* < 0.02) detected by Fisher’s exact test [31] in the total monthly number of new-born boys with CP (C), respectively

**Fig. 5:**
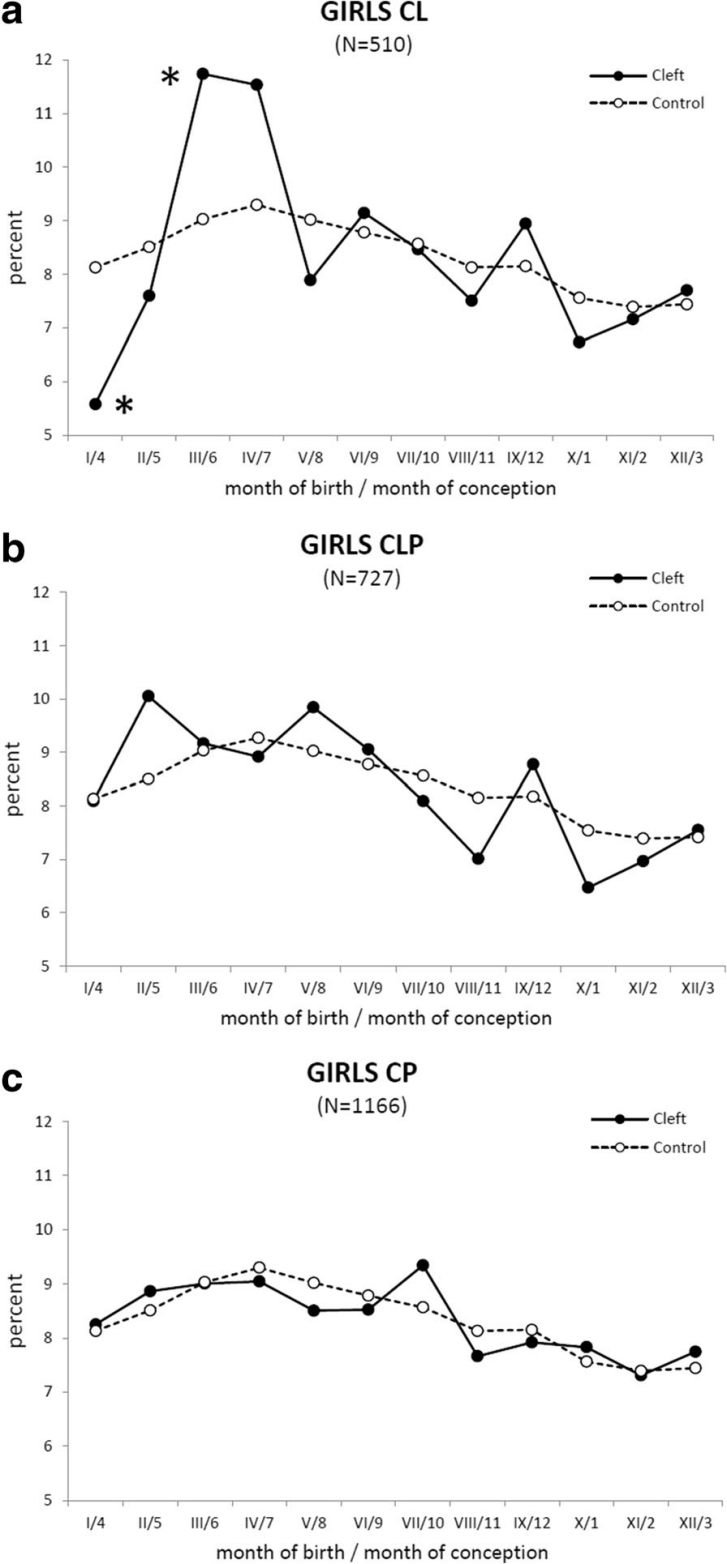
The mean daily number of new-born girls in each month. The mean daily number (MDN) in the percent of new-born girls with an orofacial cleft (solid line) is compared with the control group of girls (dashed line). **a** The girls with cleft lip and jaw (CL). **b** The girls with cleft lip and palate (CLP). **c** The girls with cleft palate (CP). Axis x: Roman digit - month of delivery, Arabic numeral - month of conception. Asterisk (*) shows the position of a decline or peak (A) corresponding to a statistically significant (*p* < 0.05) decrease or increase, respectively, detected by Fisher’s exact test [31] in the total monthly number of new-born girls with CL

For the purposes of graphical presentation (Figs. 3, 4, 5), the data on both the cleft and control groups were treated in the same way: for each particular month in the year, the mean monthly number of new-borns (x̄ in the Tables 1 and 2) was calculated from the total number of infants born in that month during the whole period 1964–2000 (**Σ** in the Tables 1 and 2). To avoid differences in the mean monthly numbers of new-borns caused by the different number of days per month (28, 29, 30 and 31), the mean daily number of new-borns in each month (MDN) was then calculated. To allow graphical presentation/comparison in the same graph between the high values in the control group and the low values in the cleft group, the MDN in a month was expressed as a percentage, with 100% being the sum of the respective means of all 12 months. The MDN values allowed plotting, graphical presentation and comparison of birth rates in the cleft group and the large control group together in the same graph.

## Results

### Control group of new-borns

The number of live-born children followed a typical rule; more boys than girls are born in each month, but the repetitive seasonal variation in the number of boys and girls was similar (Fig. 2a). Fig. 2b shows the annual course of the MDN of new-born girls + boys in the control group, expressed as a percentage, with 100% being the sum of the respective means of all 12 months. These data were evaluated by the Confidence interval test [33]. The grey strip represents a 95% confidence interval, which comprises the values that do not significantly differ from the mean value. The data located above or below the interval are significantly (*p* < 0.05) higher or lower than the mean value, respectively. In comparison to the mean value (Fig. 2b), the number of new-born boys and girls significantly (*p* < 0.05) increased in spring (March, April, May), corresponding to conception during the summer months (June, July, August). The significant seasonal peak was also confirmed when the mean monthly numbers of new-borns were evaluated by the seasonal variation tests [32] at *p* < 0.01: the peak day was detected on May 11, 2-month peak April–May, 3-month peak March–May, 4-month peak March–June, 5-month peak March–July and 6-month peak February–July.

In contrast, a significantly (*p* < 0.05) lower number of new-born babies were detected in autumn (October, November, December) by the Confidence interval test [33]. These terms of birth corresponded to conception during the winter (January, February, March), (Fig. 2b). The continuous decline in the new-born curves from April to November was interrupted by a small peak in September, corresponding to conception in December (Fig. 2b).

### Boys and girls with all types of orofacial clefts

In comparison to the control group, the seasonal variation tests [32] showed similar results at *p* < 0.01 for the mean monthly numbers of new-borns (boys + girls) with an orofacial cleft: annual peak date on May 13, and 2-month peak May–June, 3-month peak March–May, 4-month peak February–May, 5-month peak February–June, and 6-month peak February–July. Only boys with an orofacial cleft showed the annual peak date on May 22, 2-month peak May–June and 3-month peak May–July (*p* < 0.01). In solely girls with an orofacial cleft, the annual peak date was May 1, two-month peak March–April and three-month peak March–May (*p* < 0.01).

To compare and present the birth rates in the control and cleft groups (Tables 1 and 2) in one graph, the MDN of new-borns as expressed in percentages. The curve for the whole cleft group (boys + girls) was similar to the control group (Fig. 3a). During the year, no significant difference was found by Fisher’s exact test [31] between the total monthly numbers of boys and girls born during a period of 37 years.

Increased oscillation of the cleft group data around the control values appeared after plotting boys (Fig. 3b) and girls separately (Fig. 3c). In general, the MDN curve of boys with an orofacial cleft exhibited a seasonal course similar to the reference curve (Fig. 3b). However, one significant decrease (*p* < 0.05) was found by Fisher’s exact test [31] in the total number of boys with an orofacial cleft born in April, corresponding to conception in July and to the critical period for CL, CLP, and CP formation from August until October (compare to Fig. 3b).

The MDN of new-born girls with an orofacial cleft oscillated during the year around the control group data (Fig. 3c) without any significant difference in the total monthly numbers calculated for 37 years.

### Boys subdivided according to the cleft type

To search for the type of orofacial cleft responsible for the significant decrease in the total monthly number of new-born boys observed in April (Fig. 3b), we separately plotted the curves of MDN for boys with CL, CLP or CP (Fig. 4a, b, c), and the total monthly numbers of new-borns for 37 years (Table 1) were evaluated by Fisher’s exact test [31].

In comparison to the control values, the total monthly number of new-born boys with isolated CP showed a significant decrease (*p* < 0.02) in April (compare to Fig. 4c). The subsequent increased value in May did not significantly differ (*p* < 0.08) from the control group but was significantly different from April (*p* < 0.01). During 1964–2000, such a decrease in April and increase in May also repeatedly appeared in the annual monthly numbers of new-born boys with CP in 25 of the 37 years under investigation. The timing of this down-up anomaly in April–May corresponds to conception from July until August (Fig. 4c) and to the critical period for CP formation from August until October. The residences of the boys born in May with CP were concentrated mainly in Prague and in the central Bohemia district.

The total monthly numbers of boys with CLP per 37 years did not demonstrate any significant difference compared to that of the control group during the course of the year (compare to Fig. 4b).

The MDN of boys with CL oscillated around the control data with one peak (compare to Fig. 4a), corresponding to a significant increase (*p* < 0.03) in the total number of August new-borns per 37 years (**Σ** in the Table 1). Their conception was in November, and the critical period for CL formation was in December.

### Girls subdivided according to cleft type

When the sample of girls with an orofacial cleft was sub-divided into smaller groups according to cleft type, the total monthly number of girls with CP or CLP calculated per 37 years (Table 2) did not demonstrate any significant differences (Fisher’s exact test [31]) in comparison to the control group (compare to Fig. 5b, c). The MDN values of the CLP girls oscillated around the reference values, while the MDN values of the girls with CP closely tracked those of the control group (Fig. 5c**).** Fisher’s exact test [31] only detected significant differences in the total monthly numbers of girls with CL when compared to control values: a significant decrease (*p* < 0.05) in January (conception in April and critical period of CL formation in May) followed by a significant peak (*p* < 0.05) in March and non-significant peak in April (conception in June–July and critical period in July–August), (compare to Fig. 5a). During 1964–2000, such a down-up anomaly, including a decrease in January and increase in March–April, also repeatedly appeared in the annual monthly numbers of new-born girls with CP in 23 of the 37 years analysed.

## Discussion

The number of new-borns with an orofacial cleft exhibited regular seasonal variation; significantly more babies were born during March, April and May, while significantly fewer babies were born in October, November and December. Similar seasonal variation, without a significant difference from the control group, was also found in the whole group of new-borns with an orofacial cleft (CL, CLP, and CP). After subdividing the cleft patients according to gender and cleft type, there was only a significant increase in the total number of boys with CL born in August and of girls with CL born in March during a period of 37 years when compared to the control group. Conversely, a significant decrease was found in the number of boys with CP born in April and of girls with CL born in January.

### Control group of new-borns

It is a general rule that more boys than girls are born regularly. Our data on the control group are in accordance with this rule (Fig. 2). In our country, only one anomaly has been reported; in November 1986, seven months after the Chernobyl nuclear accident and the attendant release of radiation, more than 450 new-born boys were missing. This implies that this month, for the first and only time during the last 50 years, more girls than boys were born in the Czech Republic [6, 34].

During the regular seasonal decrease in the total birth number from April to December, a small peak appeared in the curves in September [6] (see also Fig. 2). This small peak reflects conception in December. This phenomenon is called the “Christmas effect”, which has been observed in many countries, e.g., in Norway [35], the USA [36] and Croatia [37].

### Increase in the number of new-borns with CL

In comparison to the control group, we found only two significant peaks in the total monthly numbers calculated for 37 years: in girls with CL in March (critical period of cleft formation in July), and in boys with CL in August (critical period in December). No significant increase was detected in the other cleft types (CP, CLP) in comparison to the control group.

Edwards [10] has tracked the seasonal variation in the proportion of children with various abnormalities standardized against the total birth number in Birmingham. Among 17 abnormalities, the maximal seasonal incidence was found in new-borns with CL in March.

A significantly above average seasonal incidence of CL, with or without CP, exists in the United States from November through March in the region characterized by hot summers and moderate winters. This might indicate that some factors prevalent in hot summer areas are involved in the malformation process [12].

In Finland, Rintala et al. [15] have observed seasonal variation in the number of new-borns with CL and CLP, with a peak in April, but no seasonal variation in the number of new-borns with CP.

### Decrease in the number of new-born boys with CP and girls with CL

We found a significant decrease in the number of boys with an orofacial cleft in April. More detailed analysis revealed that this overall decrease is caused by a specific decrease in the number of boys with CP. However, this decrease did not result from a premature delivery of the missing children, since the decline was not preceded (compensated) by a peak in March. The absence of a portion of boys with CP can be explained from either a positive aspect - the CP did not arise, or from a negative aspect – the missing boys with CP were aborted. It is known that after prenatal exposure to a strong harmful factor, the number of malformed new-borns may decrease [38] as a result of their prenatal abortion (for review see) [39–41]. This effect concerns mainly the male fraction [6, 34, 42–45].

The decrease in the mean number of CP boys in April was followed by an increase in May (Fig. 4). Such a down-up anomaly repeatedly appeared in 25 of the 37 years under investigation. Birth dates in April or May correspond to a critical period for cleft origin during August–September or September–October, respectively. Hypothetically, both the recurrent decrease in April and increase in May might be caused by an injurious factor, which is strong in the first case, with a recordable lethal effect, and which is no longer sufficiently strong in the latter case as to result in prenatal death, but strong enough to increase the number of malformations.

The decrease in the number of girls with CL in January and the subsequent increase in March can be interpreted in a similar way (Fig. 5).

Future studies should focus on elucidating the above-mentioned down-up anomalies.

### Putative harmful factors

In the present study, the timing of the effect of putative harmful factors was considered with regard to the critical period of cleft formation (Fig. 1) and the fact that both a significant decrease or increase in the number of clefts can signal prenatal damage of the embryos – death or malformation, respectively (see above). Seasonal variation in the number of inborn defects may indicate exposure of the mother to a harmful environmental agent (e.g., climatic changes, infections, dietary habits) whose presence varies through the year [46]. Influenza and other respiratory viral infections (common cold) exhibit seasonality from early autumn to spring [47, 48]. Infectious diseases accompanied by fever and/or drug intake in a pregnant woman have been reported as important risk factors for the development of an orofacial cleft in the embryo, if such factors are present during the first trimester of gravidity [11, 20, 49–53]. Regarding the critical period of cleft formation, seasonal respiratory viral infections might contribute as risk factors to the increase in the number of new-born boys with CP in May (critical period in September – October), (Fig. 4). The significant increase in the number of new-born boys with CL in August corresponding to a critical period in December (Fig. 4) might be related to autumn respiratory infections and/or psychological stress experienced by pregnant mothers at Christmas time.

The minimum and maximum values (the abovementioned down-up anomaly) in the numbers of girls with CL born in January and March, respectively, corresponded to conception during April to June (Fig. 5) and the predicted critical period for CL formation from May to July. The number of boys with CP reached minimal and maximal values in April and May, respectively, meaning that conception took place during July to August (Fig. 4) and that the critical period for CP formation occurred from August to October. Taken together, these results indicate that the abovementioned boys and girls passed the critical period of cleft formation during May to October. From May to October, there are factors that could act either individually or in combination to impair developing embryos. This warm season is mainly characterized by high temperatures, sunshine and increased levels of UV radiation, agricultural pollutants, and ozone concentrations. A correlation between the incidence of CL in girls and the intensity of UV light has been reported, and conception in winter has been recommended as a preventive measure against CL formation [20]. There is already extensive evidence of a wide spectrum of harmful health effects resulting from air pollution, including ozone levels [54]. Outdoor exposure to air ozone during the first two months of pregnancy may increase the risk of orofacial clefts [55]. Ozone is a secondary pollutant generated by photochemical reaction between volatile organic compounds (VOC) from biogenic and anthropogenic sources, NOx (produced mainly by traffic) and solar radiation; this reaction becomes more intense with increasing outdoor temperature [54, 55].

In the Czech Republic, the ground-level ozone exhibits periodic seasonal variation with the highest values observed from April to September and minimum values from November to February [56] (see Additional file 1). Similar seasonal variation is exhibited by the values of UV radiation [57] and outdoor temperature [58], (see Additional files 2 and 3). Collectively, these data suggest that the outdoor temperature, intensity of UV radiation, and levels of ground ozone, all of which reach maximum values during the warm season, might act as harmful environmental factors implicated in seasonal changes in the birth rate of babies with an orofacial cleft. In addition to the abovementioned environmental factors of the warm season, psychological stress associated with the summer holiday might initiate the stress response, including the elevation of corticoids [59] in the maternal organism. Corticoids are known to induce orofacial clefts experimentally [60, 61].

### Study strengths and limitations

The strength of the study consists of analyses of a large sample of cleft patients (5619) collected over 37 years. This sample comprises all children born with an orofacial cleft in the Bohemia region of the Czech Republic during 1964–2000. The register is complete thanks to the centralized multidisciplinary treatment of the patients in the Cleft Centre at the Plastic Surgery Clinic, Prague, Czech Republic.

The register at the clinic naturally includes only live-born children, which is why we had to use national data on live births only.

The presently used sample of the birth rate in the Bohemia region contains all live births (3,080,891) during the period 1964–2000, including prematurely born children and children with major inborn anomalies - data on the premature births or major birth defects were not available. Therefore, we used the sample of cleft patients without further selection criteria.

Since the mean incidence of all major birth defects in the children born in our country is 340.90/10,000 [62], we assume their inclusion in our control group should have no potential impact on the seasonality of the birth-rate data. Nevertheless, for the evaluation of the birth rate in the cleft group, the control group was formed by subtracting the cleft patients from the total number of live new-borns to obtain the control sample only including the new-borns without an orofacial cleft.

With regard to the prematurely born children, some misclassification of conceptions might occur by the inclusion of premature birth. However, such a misclassification could not significantly influence the results; the results are based on large sets of cumulative data for 37 years. This is documented by the regular course of the curves in the control group (Fig. 2). Furthermore, the putative misclassification would similarly concern all control and cleft patient groups.

In the groups of cleft patients, a misclassification could not explain the significant decrease in the number of boys with an orofacial cleft in April, since this decrease was not compensated by a peak in March, reflecting premature delivery of the missing children (Fig. 4). Vice versa, the significant peak in the numbers of boys and girls with CL in August and March, respectively, was followed by no decrease the following month (Figs. 4 and 5). This finding implies that these peaks do not reflect prematurely born children that are missing among new-borns a month later.

## Conclusions

The new-borns with an orofacial cleft exhibited significant differences from controls in the total monthly numbers calculated for the 37-year period (1964–2000): girls with CL during January–March and boys with CP during April–May. These dates of birth correspond to conception from April to August and to the estimated prenatal critical period for cleft formation from May to October. This latter period includes the warm season, when injurious physical, chemical and biological factors may act individually or in combination on a pregnant woman. This information should be considered in pregnancy planning. Future studies are necessary to investigate putative injurious factors during the warm season that can influence pregnancy outcome.

## Additional files

Additional file 1: Seasonal values of ground ozone, UV radiation and temperature in the Czech Republic. Stations with the highest values of maximum daily 8-h running average concentrations of ground-level ozone in 2011–2013. (Adapted according to [56]). (TIF 15548 kb)

Additional file 2: Seasonal values of UV radiation in the Czech Republic. The annual cycle of variability of the UV-ERY model (erythemally weighted solar radiation) irradiance related to variability of the total column ozone. Heavy solid line: model UV-ERY irradiance for the mean 1962–90 total ozone concentration; circles: model UV-ERY irradiance for the mean 1991–97 total ozone concentration. Thin dashed line: solar zenith angle at noon. (Adapted according to [57]). (TIF 11559 kb)

Additional file 3: Seasonal values of the air temperature in the Czech Republic. Mean monthly air temperatures in °C in the territory of the Czech Republic in three 30-yr periods: 1961–1990 red line, 1971–2000 brown line, 1981–2010 green line. (Adapted according to [58]). (TIF 11858 kb)

## Abbreviations

CL: cleft lip
CLP: cleft lip and palate
CP: cleft palate
MDN: mean daily number
UV: ultraviolet radiation
UV-ERY: erythemally weighted solar radiation

## References

[1] Condon RG (1991). Birth seasonality, photoperiod, and social change in the Central Canadian Arctic. Hum Ecol.

[2] Bronson FH (1995). Seasonal variation in human reproduction: environmental factors. Q Rev Biol.

[3] Lam DA, Miron JA (1994). Global patterns of seasonal variation in human fertility. Ann N Y Acad Sci.

[4] Cowgill UM (1964). Recent variations in the season of birth in Puerto Rico. Proc N A S.

[5] Bobak M, Gjonca A (2001). The seasonality of live birth is strongly influenced by socio-demographic factors. Hum Reprod.

[6] Peterka M, Peterkova R, Likovsky Z (2007). Chernobyl: relationship between number of missing newborn boys and the level of radiation in the Czech regions. Environ Health Perspect.

[7] Castilla EE, Orioli IM, Lugarinho R (1990). Monthly and seasonal variations in the frequency of congenital anomalies. Int J Epidemiol.

[8] Caton AR (2012). Exploring the seasonality of birth defects in the New York state congenital malformations registry. Birth Defects Res A Clin Mol Teratol..

[9] Slater BC, Watson GI, Mcdonald JC (1964). Seasonal variation in congenital abnormalities. Preliminary report of a survey conducted by the research committee of council of the college of general practitioners. Br J Prev Soc Med.

[10] Edwards JH (1961). Seasonal incidence of congenital disease in Birmingham. Ann Hum Genet.

[11] Coupland MA, Coupland AI (1988). Seasonality, incidence and sex distribution of cleft lip and palate births in Trent region, 1973-1982. Cleft Palate J..

[12] Wehrung DA, Hay S (1970). A study of seasonal incidence of congenital malformations in the United States. Brit J Prev Soc Med.

[13] Elliott RF, Jovic G, Beveridge M (2008). Seasonal variation and regional distribution of cleft lip and palate in Zambia. Cleft Palate Craniofac J..

[14] De la Vega A, Martinez E (2006). Seasonal variation in the incidence of cleft lip and palate based on the age of conception. P R Health Sci J.

[15] Rintala A, Pönkä A, Sarna S (1983). Cleft lip and palate in Finland in 1948-75: correlations to infections, seasonal and yearly variations. Scand J Plast Reconstr Surg.

[16] Yin J, Yin N, Liu L (2013). Seasonal variation in birth months of patients with oral-facial clefts. J Craniofac Surg.

[17] Siffel C, Alverson CJ, Correa A (2005). Analysis of seasonal variation of birth defects in Atlanta. Birth Defects Res A Clin Mol Teratol.

[18] Amidei RL, Hamman RF, Kassebaum DK (1994). Birth prevalence of cleft lip and palate in Colorado by sex distribution, seasonality, race/ethnicity, and geographic variation. Spec Care Dentist.

[19] Gregg TA, Leonard AG, Hayden C (2008). Birth prevalence of cleft lip and palate in Northern Ireland (1981 to 2000). Cleft Palate Craniofac J..

[20] Krost B, Schubert J (2006). Influence of season on prevalence of cleft lip and palate. Int J Oral Maxillofac Surg.

[21] Brender JD, Weyer PJ. Agricultural compounds in water and birth defects.Curr Environ Health Rep 2016;3(2):144–152. doi: 10.1007/s40572-016-0085-0. Review. 10.1007/s40572-016-0085-0 27007730

[22] Sabbagh HJ, Alamoudi NM, Abdulhameed FD, Innes NP, Al-Aama JY, Hummaida T, Almalik M, El Derwi DA, Mossey PA (2016). Environmental risk factors in the etiology of nonsyndromic orofacial clefts in the western region of Saudi Arabia. Cleft Palate Craniofac J.

[23] Schutte BC, Murray JC (1999). The many faces and factors of orofacial clefts. Hum Mol Genet.

[24] Chung MK, Lao TT, Ting YH (2013). Environmental factors in the first trimester and risk of oral-facial clefts in the offspring. Reprod Sci.

[25] Czeizel AE, Tóth M, Rockenbauer M (1996). Population-based case control study of folic acid supplementation during pregnancy. Teratology.

[26] Botto LD, Ericson JD, Mulinare J (2002). Maternal fever, multivitamin use, and selected birth defects: evidence of interaction?. Epidemiology.

[27] Van Rooij IA, Ocké MC, Straatman H (2004). Periconceptional folate intake by supplement and food reduces the risk of nonsyndromic cleft lip with or without cleft palate. Prev Med.

[28] Fogh-Andersen P (1942). Inheritance of harelip and cleft lip.

[29] Peterka M, Peterkova R, Halaskova M, Tvrdek M, Fara M, Likovsky Z (1996). Sex differences in the incidence of orofacial clefts and the question of primary prevention in families with genetic risk. Acta Chir Plast.

[30] Czech Statistical Office. http://(www.czso.cz).

[31] Dixon WJ, Massey FJ (1969). Introduction to statistical analysis.

[32] WinPepi Portal . Version 11.65. http://www.brixtonhealth.com/.

[33] Social Science Statistics. http://www.socscistatistics.com/Default.aspx.

[34] Peterka M, Peterková R, Likovsky Z (2004). Chernobyl: prenatal loss of four hundred male fetuses in the Czech Republic. Reprod Toxicol.

[35] Odegard O (1977). Season of birth in the population of Norway, with particular reference to the September birth maximum. Br J Psychiatry.

[36] Cesario SK (2002). The “Christmas effect” and other biometeorologic influences on childbearing and the health of women. J Obstet Gynecol Neonatal Nurs.

[37] Polasek O, Kolcić I, Vorko-Jović A (2005). Seasonality of births in Croatia. Coll Antropol.

[38] Mastroiacovo P, Spagnolo A, Marni E (1988). Birth defects in the Seveso area after TCDD contamination. JAMA.

[39] Peterka M, Havránek T, Jelínek R (1986). Dose-response relationships in chick embryos exposed to embryotoxic agents. Folia Morphol (Warsz).

[40] Peterka M, Peterkova R, Likovsky Z. Environmental risk and sex ratio in newborns. In: Nicolopoulou-Stamati P, Hens L, Howard CV, editors. Congenital Diseases and the Environment. Springer, 2007b; p.295–319.

[41] Wilson JG, Fraser FC (1977). Handbook of teratology. Vol. I. General principles and etiology.

[42] Jarrell J (2002). Rationale for the study of the human sex ratio in population studies of polluted environments. Cad Saude Publica.

[43] Jongbloet PH, Roeleveld N, Groenewoud HM (2002). Where the boys aren't: dioxin and the sex ratio. Environ Health Perspect.

[44] Nicolich MJ, Huebner WW, Schnatter AR (2000). Influence of parental and biological factors on the male birth fraction in the United States: an analysis of birth certificate data from 1964 through 1988. Fertil Steril.

[45] Ryan JJ, Amirova Z, Carrier G (2002). Sex ratios of children of Russian pesticide producers exposed to dioxin. Environ Health Perspect.

[46] Owens JR, Jones JW, Harris F (1985). Epidemiology of facial clefting. Arch Dis Child.

[47] Heikkinen T, Järvinen A (2003). The common cold. Lancet.

[48] Wat D (2004). The common cold: a review of the literature. Eur J Intern Med.

[49] Molnarova A, Brozman M, Schwanzerova I (1992). Prenatal virus infections and orofacial clefts. Bratisl Lek Listy.

[50] Peterka M, Tvrdek M, Likovsky Z (1994). Maternal hyperthermia and infection as one of possible causes of orofacial clefts. Acta Chir Plast.

[51] Saxen I (1975). Associations between oral clefts and drugs taken during pregnancy. Int J Epidemiol.

[52] Métneki J, Puhó E, Czeizel AE (2005). Maternal diseases and isolated orofacial clefts in Hungary. Birth Defects Res A Clin Mol Teratol..

[53] Wang W, Guan P, Xu W (2009). Risk factors for oral clefts: a population-based case-control study in Shenyang, China. Paediatr Perinat Epidemiol.

[54] Bernard SM, Samet JM, Grambsch A (2001). The potential impacts of climate variability and change on air pollution-related health effects in the United States. Environ Health Perspect.

[55] Hwang BF, Jaakkola JJ (2008). Ozone and other air pollutants and the risk of oral clefts. Environ Health Perspect.

[56] Czech Hydrometeorological Institute. http://portal.chmi.cz/files/portal/docs/uoco/isko/grafroc/13groc/gr13e/IV4_O3_GB.html

[57] Dubrovsky M, Zerefos CS (2000). Variability of daily and annual cycles of mean erythemal solar irradiance related to total ozone variability. Chemistry and Radiation Changes in the Ozone Layer.

[58] Strestik J, Roznovsky J, Stepanek P, Rožnovský J, Litschmann T (2014). Increase of annual and seasonal air temperatures in the Czech Republic during 1961-2010.

[59] Braun T, Challis JR, Newnham JP (2013). Early-life glucocorticoid exposure: the hypothalamic-pituitary-adrenal Axis, placental function, and Longterm disease risk. Endocr Rev.

[60] Baxter H, Fraser FC (1950). The production of congenital defects in the offspring of female mice treated with cortisone. A preliminary report. McGill Med J.

[61] Peterka M, Jelinek R (1983). Origin of hydrocortisone induced orofacial clefts in the chick embryo. Cleft Palate J.

[62] Sípek A, Gregor V, Horácek J (2007). Birth defects in the Czech Republic in the period 1994–2005—perinatology data. Ceska Gynekol.

[63] Peterka M, Hrudka J, Tvrdek M (2012). Extension of orofacial cleft size and gestational bleeding in early pregnancy. Acta Chir Plast.

