# Supplementary figures and images for: Long-term significant seasonal differences in the numbers of new-borns with an orofacial cleft in the Czech Republic – a retrospective study
Source: BMC Pregnancy Childbirth. 2018 Aug 28;18:348. doi: 10.1186/s12884-018-1981-0 (PMC6114744; doi:10.1186/s12884-018-1981-0)

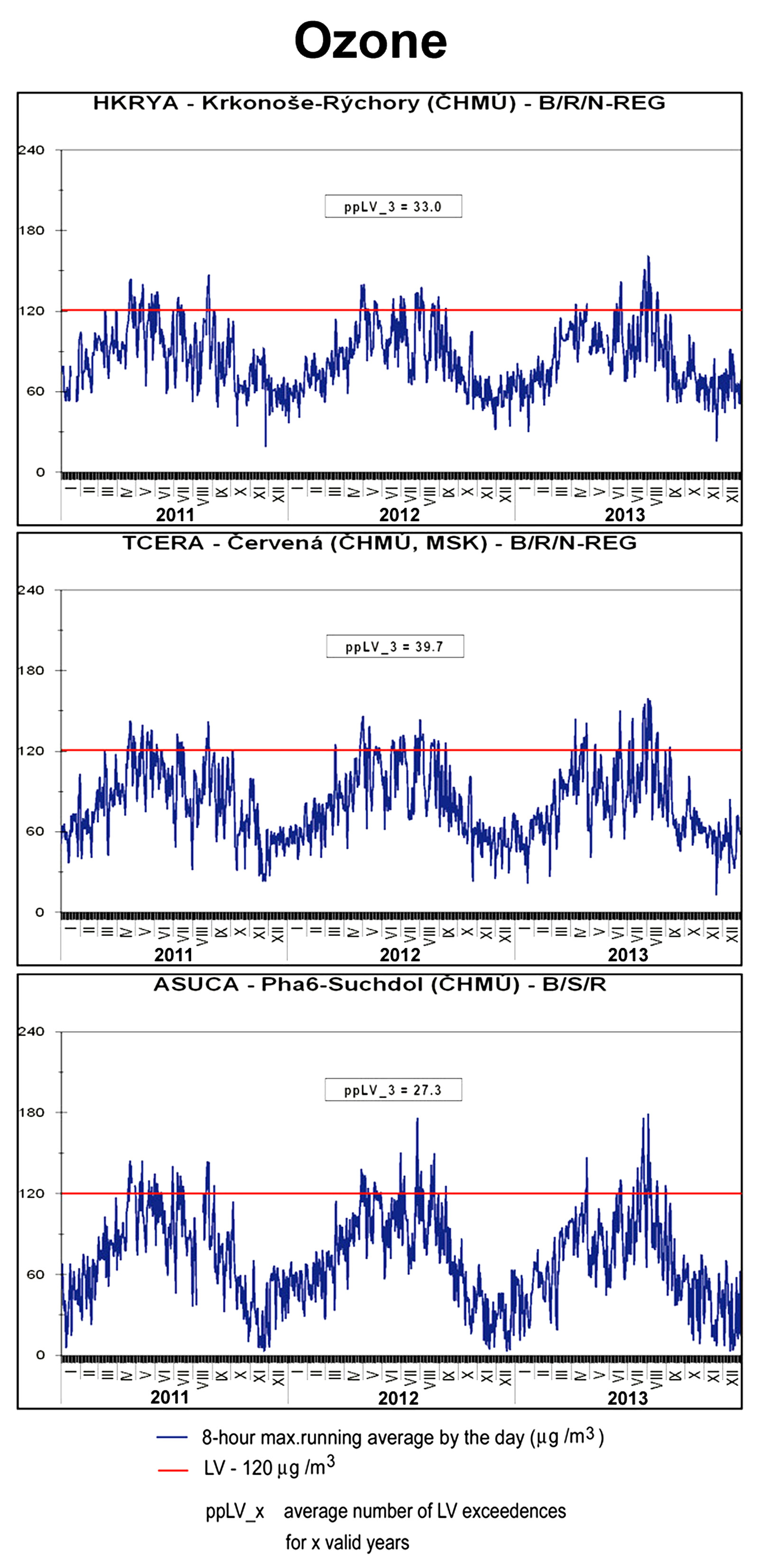

Supplement: Supplementary file 1 — Seasonal values of ground ozone, UV radiation and temperature in the Czech Republic. Stations with the highest values of maximum daily 8-h running average concentrations of ground-level ozone in 2011–2013. (Adapted according to [56]). (TIF 15548 kb) [file 12884_2018_1981_MOESM1_ESM.tif]

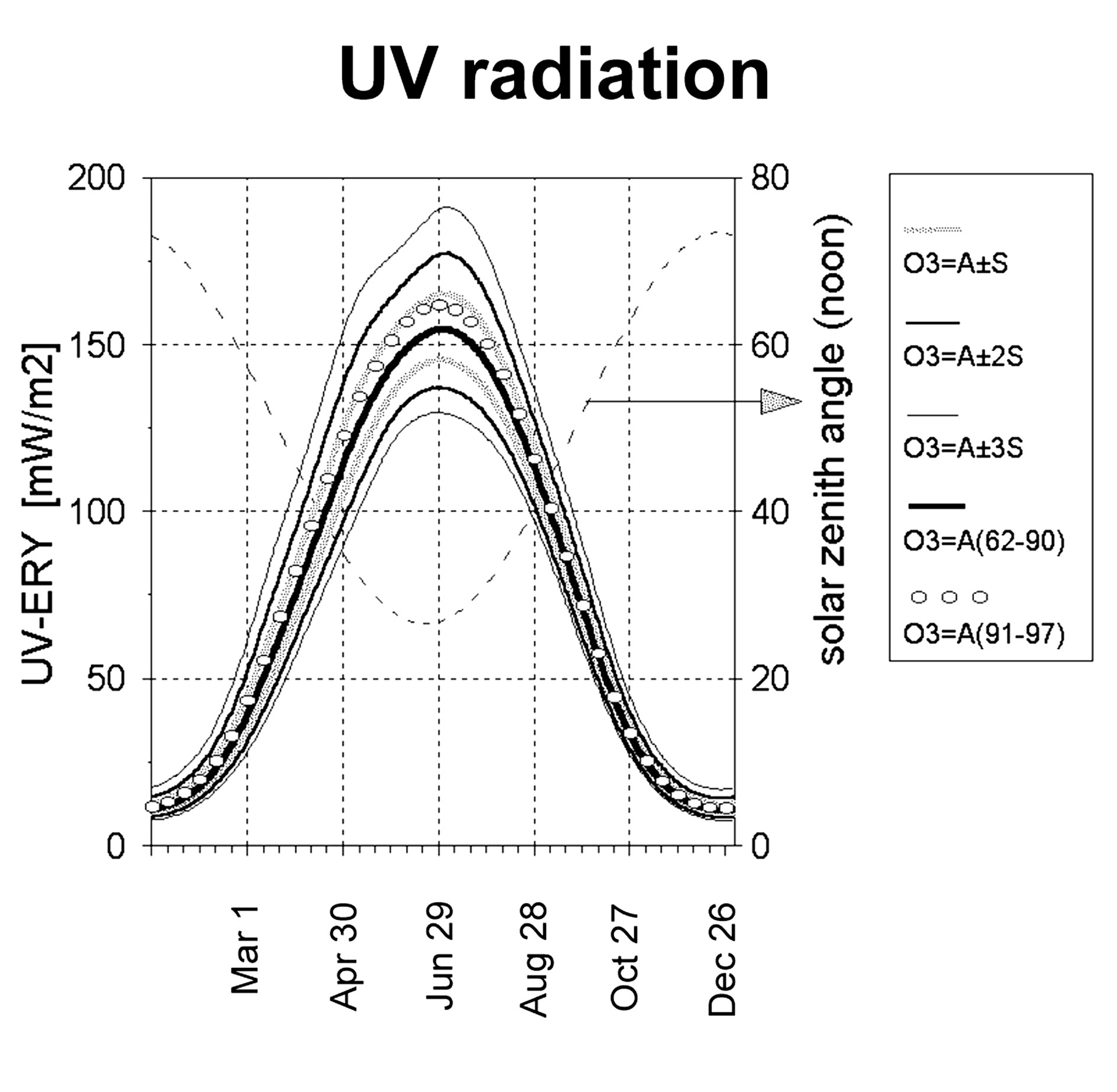

Supplement: Supplementary file 2 — Seasonal values of UV radiation in the Czech Republic. The annual cycle of variability of the UV-ERY model (erythemally weighted solar radiation) irradiance related to variability of the total column ozone. Heavy solid line: model UV-ERY irradiance for the mean 1962–90 total ozone concentration; circles: model UV-ERY irradiance for the mean 1991–97 total ozone concentration. Thin dashed line: solar zenith angle at noon. (Adapted according to [57]). (TIF 11559 kb) [file 12884_2018_1981_MOESM2_ESM.tif]

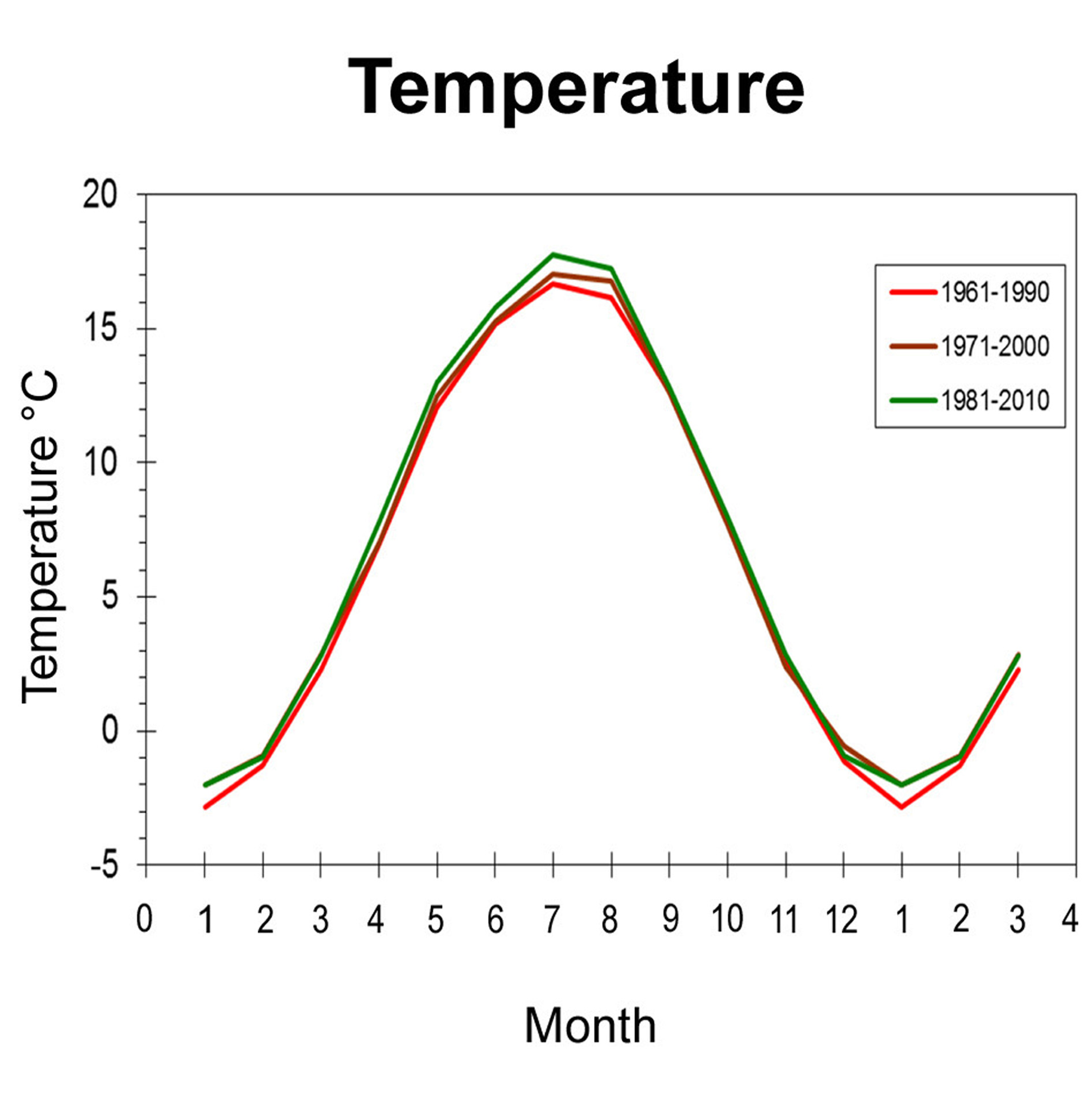

Supplement: Supplementary file 3 — Seasonal values of the air temperature in the Czech Republic. Mean monthly air temperatures in °C in the territory of the Czech Republic in three 30-yr periods: 1961–1990 red line, 1971–2000 brown line, 1981–2010 green line. (Adapted according to [58]). (TIF 11858 kb) [file 12884_2018_1981_MOESM3_ESM.tif]
